# Characteristics and Risk Factors of Cytokine Release Syndrome in Chimeric Antigen Receptor T Cell Treatment

**DOI:** 10.3389/fimmu.2021.611366

**Published:** 2021-02-23

**Authors:** Zhiling Yan, Huanxin Zhang, Jiang Cao, Cheng Zhang, Hui Liu, Hongming Huang, Hai Cheng, Jianlin Qiao, Ying Wang, Yan Wang, Lei Gao, Ming Shi, Wei Sang, Feng Zhu, Depeng Li, Haiying Sun, Qingyun Wu, Yuekun Qi, Hujun Li, Xiangmin Wang, Zhenyu Li, Hong Liu, Junnian Zheng, Wenbin Qian, Xi Zhang, Kailin Xu

**Affiliations:** ^1^ Department of Hematology, The Affiliated Hospital of Xuzhou Medical University, Xuzhou, China; ^2^ Key Laboratory of Bone Marrow Stem Cell, Blood Diseases Institute, Xuzhou Medical University, Xuzhou, China; ^3^ Medical Center of Hematology, Xinqiao Hospital, State Key Laboratory of Trauma, Burn and Combined Injury, Army Medical University, Chongqing, China; ^4^ Institute of Hematology, Zhejiang University, Hangzhou, China; ^5^ Department of Hematology, The Affiliated Hospital of Nantong University, Nantong, China; ^6^ Blood Diseases Institute, Xuzhou Medical University, Xuzhou, China; ^7^ Cancer Institute, Xuzhou Medical University, Xuzhou, China; ^8^ Jiangsu Center for the Collaboration and Innovation of Cancer Biotherapy, Cancer Institute, Xuzhou Medical University, Xuzhou, China; ^9^ Center of Clinical Oncology, The Affiliated Hospital of Xuzhou Medical University, Xuzhou, China

**Keywords:** cytokine release syndrome, chimeric antigen receptor T cell, acute lymphocyte leukemia, lymphoma, multiple myeloma

## Abstract

Clinical trials have confirmed that chimeric antigen receptor (CAR) T cell therapies are revolutionizing approaches for treating several relapsed or refractory hematological tumors. Cytokine release syndrome (CRS) is an adverse event with high incidence during CAR-T treatment. A further understanding of the characteristics and related risk factors of CRS is important for effective management. A total of 142 patients with relapsed or refractory acute lymphocyte leukemia (ALL), lymphoma, or multiple myeloma (MM) received lymphodepletion chemotherapy followed by infusion of CAR-T cells. The characteristics of CRS at different time points after treatment were monitored and risk factors were analyzed. The incidence of CRS for ALL, lymphoma, and multiple myeloma were 82%, 90%, and 90% respectively. Fever was observed on a median of day 3 for ALL, day 1 for lymphoma, and day 8.5 for MM after CAR-T cell infusion, and the duration was different between grade 1–2 CRS and grade 3–5 CRS. Disease types, peak concentration of IL-6, and CRP were associated with CRS. For patients with ALL, numbers of lymphoblast in bone marrow before lymphodepletion, peak concentration of IL-6, and CRP were independent risk factors of CRS. Clinical stage of lymphoma patients and high tumor burden in marrow of MM patients were independent risk factors of CRS. In conclusion, the characteristics and risk factors of CRS in different B-cell hematological tumors are different and should be managed individually during CAR-T cell therapy.

## Introduction

Clinical trials have confirmed that CAR-T has become an important approach for treating relapse or refractory hematological tumors (1–3). However, adverse events in CAR-T treatment are a major obstacle that can even cause death. CRS is one of the adverse events with high incidence in CAR-T cell treatment (4). According to the published data, more than 54–91% of patients may develop different grades of CRS during treatment (5). Therefore, it is important to improve the prognosis through evaluation of severity and timely intervention of CRS. However, currently available diagnostic criteria and severity grading systems of CRS are based on clinical manifestations that may delay the diagnosis and treatment of CRS (6). Therefore, a deep understanding of the characteristics of CRS and related risk factors has great clinical significance for effective management.

Several groups have tried to explore and identify risk factors of CRS (1, 7, 8), especially using laboratory biomarkers to predict severe CRS. The data showed that a 250-fold increase of single cytokine or a 75-fold increase of two cytokines suggests severe CRS (1). IL-1 increases earlier than IL-6 and blocking IL-1 also abolishes both CRS and neurotoxicity, resulting in substantially extended leukemia-free survival (9). Several studies (8, 10–12) also found that tumor burden, intensity of lymphodepletion chemotherapy, CAR-T cell dose, and thrombocytopenia were risk factors of CRS. In addition, patients with severe CRS subsequently have elevated endothelial cell activation markers such as Angiopoietin-2 and von Willebrand Factor before lymphodepletion chemotherapy (8). These studies have important implications for predicting the occurrence and development of CRS. However, these characteristics and risk assessment of CRS are based on CD19 CAR-T cell therapy. Our clinical experience (13, 14) and more and more recently published data show that the onset time, clinical characteristics, and severity of CRS are different among MM, ALL, and lymphoma (1–3). It is suggested that, in addition to CRS grading, the characteristics and risk factors of CRS should also be taken into consideration in the treatment of different B-cell hematological tumors.

Therefore, we analyzed the characteristics and risk factors of CRS in four centers in China. We observed and analyzed the available factors in patients with different B-cell hematological tumors to provide direct and reliable indicators for clinicians to manage CRS.

## Patients and Methods

### Study Design and Patient Information

A total of 142 patients with B cell hematologic malignancies received CAR-T cell treatment in four clinical centers of China. All clinical studies have been approved by the ethics committee and registered with the Chinese Clinical Trial Registration Center, respectively (ChiCTR-OIC-16008291, ChiCTR-OOC-16008447 and NCT03258047). All eligible patients were enrolled according to inclusion and exclusion criteria of the clinical studies. Patients were eligible if they were 18–69 years of age and had confirmed relapsed or refractory MM, ALL, or lymphoma, a Karnofsky Performance Score of 50 points or more, and a life expectancy of more than 12 weeks without active infections and serious liver, kidney, heart, and other diseases. Female patients who had negative serum HCG without pregnancy planned within 6 months after treatment were included. Patients with a history of mental illness, a high degree of allergies, or severe allergies (especially those who are allergic to IL-2) were excluded.

### Pretreatment and CAR-T Cell Infusion

The lymphodepletion chemotherapy included FC [fludarabine (three daily doses of 30mg/m²) and cyclophosphamide (one daily dose of 750 mg/m²)] cyclophosphamide alone or no pretreatment for 2 patients who were prior transplant. Infused CAR-T cells included anti-CD19 CAR-T cell, anti-BCMA CAR-T cell, and anti-CD20 CAR-T cell. Glucocorticoids were not used to prevent allergic reactions prior to infusion. Due to the risk of arrhythmia, cardiac monitoring was performed from the time of CAR-T cell infusion until no sign of CRS.

### Evaluation of Adverse Events and Serum Biomarkers

The adverse events were evaluated using the cytokine release syndrome evaluation criteria proposed by Lee and colleagues (6) and Common Terminology Criteria for Adverse Events version 4.0 (14). Clinical manifestations and vital signs associated with CRS were recorded at any time during treatment. Peripheral blood was collected to detect IL-6, ferritin, C-reactive protein (CRP), blood cells, creatinine, liver transaminase, bilirubin, and coagulation profiles before pretreatment and every 2 days after CAR-T cell infusion. If the patient had heart palpitations, myocardial enzymes, electrocardiogram, and troponin were measured. Complete blood cell count and chemistry panel were performed more than one time per day for patients at high risk of severe CRS and/or CRES, or those with a high tumor burden.

### Statistical Methods

Descriptive statistics (median/IQR/range, count, and percent) are reported for key variables. Fisher’s exact test, Kruskal-Wallis test, and Nemenyi test was used to compare categorical (gender, transplant, disease type, CAR-T cell dose, costimulatory molecules, species of scFv, risk stratification, clinical stage, type of light chain, and ISS stage) and continuous variables (age, blast cell, peak concentration of IL-6 and CRP, CD4/CD8, and β2-MG) among Non-CRS, grade 1–2 CRS, or 3–5 CRS. Ordinal logistic regression was used to estimate the risk factors of the occurrence of CRS. Tests were generally performed at a significance level of 0.05. All p-values reported were two-sided without adjustments for multiple comparisons. The time points of measuring biomarkers were chosen based on the clinical trial protocol and the need of clinical management. Statistical analyses were performed using SPASS (version 22.0).

## Results

### Patient Treatment Characteristics and Response

A total of 142 patients with relapsed or refractory hematology malignancies were included in the analyses. Eighty-seven (61.3%) patients were males and 55 (38.7%) females. The median age was 45 (IRQ=24–59). Fifty-five (55.7%) patients with ALL (5 Ph-positive ALL and 14 received allogeneic hematopoietic stem cell transplantation previously) received anti-CD19 CAR-T cell, and 25 patients with MM, including 11 (7.7%) type IgG, 5 (3.5%) IgA, 5 (3.5%) light chain, and 4 (2.8%) other types. Seven (28.0%) MM patients were in stage II and 18 (72.0%) in stage III. All MM patients received a combination of humanized anti-CD19 and anti-BCMA CAR T cells treatment. There were 62 patients with lymphoma, including 47 (33.1%) patients with diffuse large B-cell lymphoma, 5 (3.5%) follicular cell lymphoma, 2 (1.4%) mantle cell lymphoma, and 8 (5.6%) other types of B-cell lymphoma. Forty (28.2%) patients with lymphoma received CD19+CD20 CAR-T and 22(15.5%) received CD19 CAR-T (**Table 1**).

**Table 1:** Characteristics of Patients (n=142).

Variables	All patients (%)
**Gender**	
M	87 (61.3%)
F	55 (38.7%)
**Age, median (IRQ)**	45 (24–59)
**Disease**	
**ALL**	55 (38.7%)
Ph positive	
Yes	5 (3.5%)
No	50 (35.2%)
Prior Transplant	
Yes	14 (9.9%)
No	41 (28.9%)
**MM**	25 (17.6%)
MG	
IgG	11 (7.7%)
IgA	5 (3.5%)
Light chain	5 (3.5%)
Other malignant plasmacyte disease	4 (2.8%)
Disease stage at diagnosis (ISS staging)	
II	6 (4.2%)
III	18 (12.7%)
**Lymphoma**	62 (43.7%)
NHL	
DLBCL	47 (33.1%)
FL	5 (3.5%)
MCL	2 (1.4%)
Other BL	8 (5.6%)
**Target of CAR-T cell**	
ALL	
CD19	55 (38.7%)
MM	
CD19+BCMA	25 (17.6%)
Lymphoma	
CD19	22 (15.5%)
CD19+CD20	40 (28.2%)

The overall response rates (ORR) of ALL, lymphoma, and myeloma were 85%, 70%, and 95.2%, respectively. The complete response (CR) was 85% in ALL patients, and the CR and partial response (PR) was 30% and 40% in patients with lymphoma, respectively. In patients with MM, CR, very good partial response (VGPR), and PR were 45%, 23%, and 20% respectively at one month after CAR-T cell infusion (**Figure S1**).

### The Incidence of CRS and Characteristics

**Figure 1:**
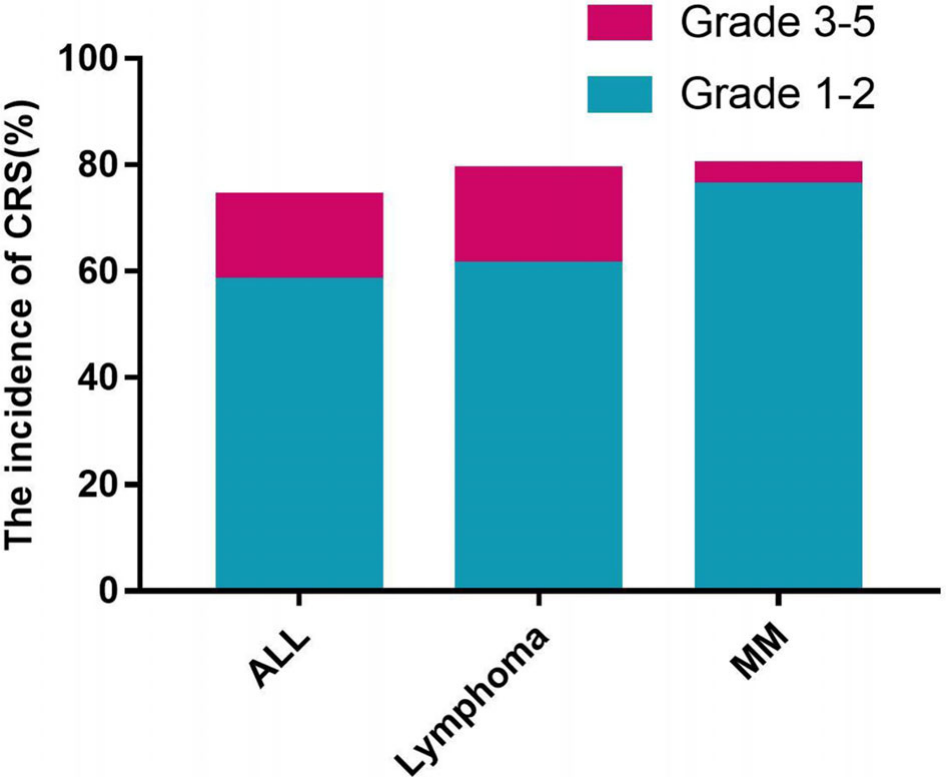
The incidence of CRS.

The CRS incidence of ALL, lymphoma, and MM were 82%, 90%, and 90%, respectively. However, the severity of CRS was different among MM, ALL, and lymphoma. Grade 1–2 and grade 3–5 CRS were found in 33 (60%) and 11 (20%) patients with ALL respectively. In patients with lymphoma, grade 1–2 CRS were observed in 45 of 62 (72.6%) and grade 3–5 in 10 of 62 (16.1%). But only one patient with MM encountered grade 3 CRS and most patients had grade 1 or 2 CRS (**Figure 1**). One patient with ALL died of heart failure resulting from the CRS-related myocarditis. Three patients with lymphoma and one patient with ALL had developed gastrointestinal bleeding.

Fever was the most common sign of CRS. In all patients, there was no difference in the onset time of fever between grade 1–2 and grade 3–5 CRS. But there was a difference among patients with different diseases. The median onset time of fever was day 3 (IRQ, day 0–7) in ALL patients, day 1 (IRQ, day 0–5) in patients with lymphoma, and day 8.5 (IRQ, day 1.75–12.75) in MM patients. The onset time of fever was different between MM and ALL (*p*=0.0044), or MM and lymphoma (*p*=0.0002), but no difference between ALL and lymphoma (*p*=0.5549). Further analysis according to the disease type and CRS level showed that the median onset time of fever in ALL patients with grade 1–2 and grade 3–5 CRS were day 4 (range day 0–10) and day 1 (range day 0–7) respectively and there was no difference. Fever occurred on a median of 1.5 days (range, 0–16 days) after CAR-T cell infusion in lymphoma patients with grade 1–2 CRS and without difference compared to patients with grade 3–5 CRS. Only one patient with MM developed grade 3 CRS, and the onset time of fever was earlier than those with grade 1–2 CRS (**Table 2**).

**Table 2:** Characteristics of fever in patients with CRS.

Patients	Grade 1–2 CRS	Grade 3–5 CRS	*P* value*
All patients, Median (range)			
Initial time of fever, day	3 (0–16)	1 (0–7)	0.062
Duration of fever, day	4 (1–36)	8 (2–32)	0.007
Peak of fever, °C	39.4 (37.7–41.9)	40 (37.9–40.5)	0.02
ALL, Median (range)			
Initial time of fever, day	4 (0–10)	1 (0–7)	0.33
Duration of fever, day	5 (1–36)	8 (2–20)	0.094
Peak of fever, °C	39.8 (38–41.9)	40.1 (39.6–40.5)	0.269
Lymphoma, Median (range)			
Initial time of fever, day	1.5 (0–16)	1 (0–7)	0.273
Duration of fever, day	4 (1–18)	5 (3–32)	0.194
Peak of fever, °C	39.1 (37.7–40.5)	39.3 (37.9–40.3)	0.654
MM, Median (range)			
Initial time of fever, day	9 (0–15)	4	–
Duration of fever, day	4 (1–11)	11	–
Peak of fever, °C	39.3 (38.1–41)	40.4	–

*Two-sided P-values calculated based on Kruskal-Wallis test.

The duration of fever in all patients was significantly different between grade 1–2 and grade 3–5 CRS (*p*=0.007). However, there was no statistical difference in the median duration of fever among ALL [3 days (0–7 days)], lymphoma [5 days (3–8 days)], and MM [4 days (3–8 days)]. The peak temperature of fever was different between grade 1–2 and grade 3–5 CRS (*p*= 0.02) and no difference among different diseases [ALL: 40°C (39.15–40.5°C) vs lymphoma: 39.4°C (38.8–39.8°C) vs MM: 39.1°C (38.8–39.7°C)] (**Table 2**).

Changes of peak concentration of IL-6 and CRP in serum were consistent with severity of CRS. There was difference of IL-6 concentration in serum between non-CRS and grade 3–5 CRS patients with ALL on days 7 and 10 and without differences among them at other time points. There were no differences in CRP among different B cell tumors patients with non-CRS, grade 1–2 CRS, and grade 3–5 CRS at specific time points (day 0, 3, 7…after CAR-T cells infusion). However, the peak concentration of IL-6 and CRP during CRS were significantly higher than the baseline (**Table S1**, **Figure S2**).

### Clinical Factors Related to CRS

We analyzed age, gender, prior transplantation, disease type, CAR-T cell dose, and costimulatory molecules of all patients, separately. However, there were no differences between patients with grade 1–2 CRS and grade 3–5 CRS, except for disease type (ALL versus MM, *p*=0.049). The peak concentration of IL-6 (*p*=0.000) and CRP (*p*=0.001) was different among the patients with non-CRS, grade 1–2, or grade 3–5 CRS. Further analysis showed that there was statistical difference in the peak concentration of IL-6 between the patients with Non-CRS and grade 1–2 CRS (*p*=0.00), Non-CRS and grade 3–5 CRS (*p*=0.00), or grade 1–2 and 3–5 CRS (*p*= 0.03). The peak concentration of CRP was different between the patients with Non-CRS and grade 1–2 CRS (*p*=0.01), Non-CRS and grade 3–5 CRS (*p*=0.00), but there was no difference between grade 1–2 and grade 3–5 CRS (*p*=0.18) (**Table 3**). In the regression model, peak concentration of IL-6 (OR: 1.001, 95% CI: 1.0–1.001) and CRP (OR: 1.011, 95% CI: 1.005–1.017) could predict CRS (**Table S2**). The concentration of IL-6 >54.95pg/ml has 81.8% specificity and 61.0% sensitivity and the concentration of CRP >88.45mg/ml has 91.3% specificity and 52.1% sensitivity for CRS (**Figure S3**, **S4**).

**Table 3:** Clinical general factors related to cytokine release syndrome, by grade **(n=119)**.

Variable	CRS Grade		Univariate
	Grade 1–2	Grade 3–5	Analysis *P value
**Gender, n (%)**			0.860
Male	61 (51.3%)	14 (11.8%)	
Female	38 (31.9%)	8 (6.7%)	
**Age, Median [IQR]**	49 (28–59)	37 (23–51.75)	0.317
**Prior Transplant**			0.510
Yes	13 (10.9%)	4 (3.4%)	
No	86 (72.3%)	18 (15.1%)	
**Disease Type, n (%)**			
ALL	33 (27.7%)	11 (9.2%)	0.049^※^
Lymphoma	45 (37.8%)	10 (8.4%)	0.163
MM	21 (17.6%)	1 (0.8%)	
**CAR-T Cell Dose**			
10^8^ or >10^8^	56 (47.1%)	15 (12.6%)	0.343
10^7^	25 (21.0%)	5 (4.2%)	0.687
10^6^ or <10^6^	18 (15.1%)	2 (1.7%)	
Costimulatory molecules			1.0
CD28	9 (7.6%)	2 (1.7%)	
4-1BB	90 (75.6%)	20 (16.8%)	

*Two-sided P-values calculated based on Kruskal-Wallis test for continuous variables, and Fisher’s Exact test for categorical variables.

^※^ALL versus MM.

### Risk Factors of CRS in Different B Cell Tumors

Each B-cell tumor has its own staging or prognosis evaluation systems. We analyze the relationship between these available factors and the occurrence of CRS.

### ALL

We analyzed a variety of clinical factors that may be associated with CRS (gender, age, transplantation, CAR-T cell dose, bone marrow tumor burden, species of CAR, costimulatory molecules, serum maximum values of IL-6 and CRP, and minimum level of CD4/CD8) separately. Univariate analysis showed that the number of blasts cells in bone marrow (*p*=0.003), serum peak concentration of IL-6 (*p*=0.001) and CRP (*p*=0.008), and minimum value of CD4/CD8 (*p*=0.028) are the influencing factors for the occurrence of CRS. These factors were further entered into an ordinal logistic regression model, and the results showed that the number of blasts in bone marrow (OR:1.034, 95% CI 1.011-1.058) was the independent risk factors for CRS (**Table 4**, **Table S3**). The number of blast cells in bone marrow >22.0% (before pretreatment) has 45.0% specificity and 90.9% sensitivity for severe CRS (**Figure S5**).

**Table 4:** Clinical factors related to cytokine release syndrome (patients with ALL, n=55).

Variable	CRS Grade			Univariate Analysis	Multivariable Analysis		
	non-CRS	Grade 1–2	Grade 3–5	*P* value	OR	95% CI	*P* value
Gender, n (%)				0.46			
Male	8 (14.5)	17 (30.9)	5 (9.1)				
Female	3 (5.5)	16 (29.1)	6 (10.9)				
Age							
Median [IQR]	17 (5–41)	15 (3–69)	25 (15–66)	0.176			
Prior Transplant				0.69			
Yes	2 (3.6)	8 (14.5)	4 (7.3)				
No	9 (16.4)	25 (45.5)	7 (12.7)				
CAR-T Cell Dose				0.29			0.794^a^
10^6^>10^7^	8 (14.5)	24 (43.6)	5 (9.1)				
10^8^ or >10^8^	3 (5.5)	9 (16.4)	6 (10.9)				
Bone Marrow Blast Cell, Median [range]							
Before FC treatment	0 (0–93)	62 (0–88)	80 (15–95)	0.003	1.034	1.011–1.058	0.004^a^
Before CAR-T infusion	0 (0–92.5)	6 (0–92)	18 (0–95)	0.053	–	–	0.758^a^
Species of scFv, n(%)				0.17			0.173^a^
humanization	4 (7.3)	22 (40.0)	8 (14.5)				
mouse	7 (12.7)	11 (20.0)	3 (5.5)				
Costimulatory molecules				0.385			0.184^a^
CD28	5 (9.1)	9 (16.4)	2 (3.6)				
4-1BB	6 (10.9)	24 (43.6)	9 (16.4)		–	–	

*Two-sided P-values calculated based on Kruskal-Wallis test for continuous variables, and Fisher’s Exact test for categorical variables.

**^※^**Ordinal Regression were performed to assess impact of baseline factors on the occurrence of CRS.

^a^The variables that enter the regression model include: Univariate Analysis (P≤0.1) or the variables that may affect the results.

### Lymphoma

We analyzed a variety of clinical factors (gender, age, risk stratification, clinical stage, CAR-T cell dose, serum peak concentration of IL-6 and CRP, CD4/CD8, etc.) that may be associated with CRS in patients with lymphoma, separately. Univariate analysis showed that gender (*p*=0.016), serum peak concentration of IL-6 (*p*=0.016), were related to the occurrence or severity of CRS. Ordinal logistic regression model showed that sex (OR:0.113, 95% CI:0.019–0.666) and clinical stage (stage IV vs stage II, OR:0.05, 95% CI: 0.03–0.926) are independent risk factors for the CRS (**Table 5**, **Table S4**).

**Table 5:** Clinical factors related to cytokine release syndrome (patients with Lymphoma, n=62).

Variable	CRS Grade			Univariate Analysis	Multivariable Analysis		
	non-CRS	Grade 1–2	Grade 3–5	*P* value	OR	95% CI	P value
**Gender**, n (%)				0.016			
Male	2 (3.2%)	35 (56.5)	9 (14.5)		0.113	0.019–0.666	0.016^a^
Female	5 (3.2%)	10 (16.1)	1 (1.6)				
Age							
Median [IQR]	51 (23–62)	52 (24–72)	43.5 (17–70)	0.571			
Risk stratification (IPI)				0.554			
High	0	12 (19.4%)	2 (3.2%)				0.969^a^
Moderate	4 (6.5%)	20 (32.3%)	6 (9.7%)				0.687^a^
Low	3 (4.8%)	13 (21.0%)	2 (3.2%)				
Clinical stage				0.058			
IV	3 (4.8%)	19 (30.6%)	9 (14.5%)		0.05	0.03–0.926	0.044^a^
III	3 (4.8%)	23 (37.1%)	1 (1.6%)				0.133^a^
II	1 (1.6%)	3 (4.8%)	0				
CAR-T Cell Dose				0.579			
10^5^>10^7^	2 (3.2%)	18 (29.0%)	2 (3.2%)				
10^8^>10^9^	5 (3.2%)	27 (43.5%)	8 (112.9%)				

*Two-sided P-values calculated based on Kruskal-Wallis test for continuous variables, and Fisher’s Exact test for categorical variables.

**^※^**Ordinal Regression were performed to assess impact of baseline factors on the occurrence of CRS.

^a^The variables that enter the regression model include: Univariate Analysis (P≤0.1) or the variables that may affect the results.

### MM

We analyzed a variety of clinical factors (gender, age, β2-MG, type of light chain, ISS stage, plasma cell number in bone marrow, peak concentration of IL-6 and CRP, and minimum value of CD4/CD8) that may be associated with CRS in patients with multiple myeloma separately. But univariate analysis showed that these factors were not related to the occurrence or severity of CRS. However, based on clinical experience, several factors (β2-MG and number of plasma cells) were entered in an ordinal logistic regression model that may be related to CRS. The results showed that the number of plasma cells in the bone marrow (OR:1.072, 95% CI:1.008–1.140) is an independent risk factor for CRS (**Table 6**, **Table S5**).

**Table 6:** Clinical factors related to cytokine release syndrome (patients with MM, n=25).

Variable	CRS Grade			Univariate Analysis	Multivariable Analysis		
	non-CRS	Grade 1	Grade 2–3	*P* value	OR	95% CI	P value
**Gender**, n (%)				0.869			
Male	3 (12.0)	4 (16.0)	4 (16.0)				
Female	2 (8.0)	7 (28.0)	5 (20.0)				
Age							
Median [IQR]	62 (51–65)	59 (53–63)	52 (46–59)	0.140			
β2-MG, n (%)	6713.5 (2413–18600)	3159 (1843–6511)	2888 (1763–12522)	0.455			0.056^a^
Type of Light chain				1.0			
Kappa	2 (8.0)	7 (28.0)	6 (24.0)				
Lambda	1 (4.0)	4 (16.0)	3 (12.0)				
Myeloma Cells in Bone Marrow,							
Median (range)	16 (8–21)	12 (2–69)	24 (3–67)	0.343	1.072	1.008-1.140	0.028^a^
ISS stage				1.0			
II	1 (4.0)	3 (12.0)	3 (12.0)				
III	4 (16.0)	8 (32.0)	6 (24.0)				

*Two-sided P-values calculated based on Kruskal-Wallis test for continuous variables, and Fisher’s Exact test for categorical variables.

**^※^**Ordinal Regression were performed to assess impact of baseline factors on the occurrence of CRS.

^a^The variables that enter the regression model include: Univariate Analysis (P≤0.1) or the variables that may affect the results.

## Discussion

CRS is one of the major complications during CAR-T cell treatment. However, current guidelines or options of management of CRS are based on data of CD19 CAR and risk assessment of CRS occurrence of different diseases (ALL, lymphoma, or MM) use the same standard or method (5, 15–18). This is not reasonable to management of CRS of patients with B-cell hematological tumors. In this *post hoc* analysis, we found that although the clinical manifestations of CRS in different diseases are similar, the characteristics and risk factors of CRS are not the same, suggesting that we need to pay more attention to the management of CRS according to disease type, instead of treating them in the same way.

In our study, there is no difference in the total incidence of CRS among patients with MM, ALL, or lymphoma. However, the incidence of severe CRS in patients with MM is significantly lower than those with ALL or lymphoma. We are not sure if this phenomenon is caused by the antigen itself, kinetics of CAR-T cell proliferation, the immune microenvironment, or others. Two patients with lymphoma and one patient with ALL were complicated with gastrointestinal bleeding during CRS. Because the general condition of the patient was very poor, we were unable to perform colonoscopy and pathology to determine the true cause of gastrointestinal bleeding, but we should pay attention to this fatal complication. One patient with ALL died of acute myocarditis. This patient first showed elevated glutamic oxaloacetic transaminase (ALT) and lactic dehydrogenase (LDH) in serum, without any other special clinical manifestations. Although glucocorticoid and IL-6 were used, the patient suddenly developed heart failure heavily and died. Therefore, for patients with myocardial damage, we should be vigilant for fatal heart failure. Therefore, to balance the possible advantages and disadvantages of intensive treatment (tochizumab, glucocorticoid, etc.), MM patients with CRS have more sufficient observation time, while patients with acute lymphoblastic leukemia and lymphoma need to be more cautious and timely, especially for patients with organ damage.

Fever is the primary manifestation of initiation of CRS in most patients. Although the fever types of different diseases were similar, the median onset time of fever in patients with lymphoma was the earliest, followed by ALL and MM. The mechanism of CRS is still unclear. According to our and other published data, the proliferation of CAR-T cell in MM patients is relatively slower than that in those with ALL (1, 3, 13, 14), which may be the cause of delayed occurrence of CRS. Another interesting phenomenon is that we, as well as other research groups, have found that the incidence of CRS is high during BCMA CAR-T cells in patients with relapsed or refractory MM, but the incidence of severe CRS is very low. Therefore, when fever occurs early after CAR-T cell infusion, CRS should be considered first for patients with acute lymphoblastic leukemia and lymphoma, and the changes of peripheral oxygen concentration, blood pressure, organ function, and blood biological markers (IL-6, CRP, and ferritin, etc.) should be monitored more frequently. However, in MM patients, the onset time of CRS related fever is relatively late, which may coexist with infection and bring more challenges to diagnosis and treatment of CRS.

Cytokines are the critical factors in the CRS (9, 19, 20), including ferritin, IL-6, CRP, TNF, interferon, IL-10, IL-1, MCP, etc. Among them, the most commonly used in the clinic are ferritin, IL-6, and CRP. Several clinical studies (8) have confirmed that the serum levels of these factors are associated with the occurrence and severity of CRS, and dynamic changes reflect the outcome of CRS. In our study, we found that the trends of these factors at the different time points (day 3, day 7, day 10…) were consistent with the occurrence or progression of CRS. However, only IL-6 levels at specific time point (day 7 and 10) were different in ALL patients between grade 3–5 CRS and non CRS. But a significant difference in the peak concentration of IL-6, ferritin, and CRP occurred among patients with different levels of CRS, indicating that the cytokines level at a specific time point does not truly reflect their trends. We also found that cytokine levels can change sharply in a few days or even hours. Therefore, we should monitor the changes of cytokines level more frequently according to the severity of CRS, rather than at specific time points.

The occurrence and severity of CRS are related to several factors, including CAR-T cells dose, proliferation of CAR-T cells, and the number of blasts in the bone marrow (8, 21). Early CRS risk assessment helps to monitor and intervene in a timely manner for patients with high-risk factors. The patient’s baseline characteristics are the most available predictor (7, 8). Our data showed that the type of disease was an important factor of the severity of CRS, and the different B-cell hematological tumors have their own predictive risk factors. Moreover, IL-6 and CRP were the independent risk factor not only for the occurrence of CRS but also for the severity of CRS. For patients with acute lymphoblastic leukemia, tumor burden was the high-risk factor for CRS. Therefore, reducing the tumor burden before CAR-T cell therapy may reduce the occurrence of CRS. Clinical stage is associated with CRS in patients with lymphoma (especially stage IV). For patients with lymphoma involving organs (small intestine, liver, lungs, etc.), while monitoring or intervening CRS, more attention should be paid to the damage (gastrointestinal bleeding, pulmonary edema, liver failure) of the involved organs. The number of abnormal plasma cells in the bone marrow is a high risk factor of CRS in patients with multiple myeloma, and reducing the tumor load as much as possible before CAR-T cell therapy may be one of the strategies to reduce CRS. This is a retrospective study. Different manufacturers and multiple combinations of CAR-T cells, sample size available for each disease, different CAR product or construct also may lead to differences in the incidence and severity of CRS. In addition, limited observational factors may also miss some factors that may affect CRS. Therefore, in the future, more rigorous clinical studies need to be designed to verify the factors that might predict CRS.

## Conclusions

The occurrence of CRS in different B-cell tumors has its own characteristics. Compared with ALL and lymphoma, severe CRS incidence in MM patients is lower and occurs later. The risk factors of CRS in different B-cell tumors are different, suggesting that individualized treatment is required in clinical practice.

## Data Availability Statement

The original contributions presented in the study are included in the article/**Supplementary Material**. Further inquiries can be directed to the corresponding authors.

## Ethics Statement

The studies involving human participants were reviewed and approved by the ethics committees of the affiliated Hospital of Xuzhou Medical University, Xinqiao Hospital, the First Affiliated Hospital of Zhejiang University, and the First Affiliated Hospital of Nantong University. The patients/participants provided their written informed consent to participate in this study.

## Author Contributions

KX, XZ, WQ, HoL, ZL, JZ, JC, and ZY designed the research. All investigators and their respective research teams recruited and followed up the patient. ZY, CZ, HuL, HZ, HH, YQ, and YiW collected and analyzed research data. ZY and HZ wrote and edited the manuscript. All authors were involved at each stage of manuscript preparation and approved the final version. All authors contributed to the article and approved the submitted version.

## Funding

The authors would like to thank the financial support provided by National Natural Science Foundation of China (81930005, 81671584, 81871263, 81830006, 81670178, 81500088), Natural Science Foundation of Jiangsu Province (BK20161178), Key Research & Development Plan of Jiangsu Province (BE2015625, BE2017639), Scientific research project of Jiangsu Province health and Family Planning Commission (Q201506), China Postdoctoral Science Foundation project (2016M591928), Jiangsu Provincial Key Medical Discipline, and The Project of Invigorating Health Care through Science, Technology and Education (NO.ZDXKA2016014).

## Conflict of Interest

The authors declare that the research was conducted in the absence of any commercial or financial relationships that could be construed as a potential conflict of interest.
