# Supplementary material for: Characteristics and Risk Factors of Cytokine Release Syndrome in Chimeric Antigen Receptor T Cell Treatment
Source: Front Immunol. 2021 Feb 23;12:611366. doi: 10.3389/fimmu.2021.611366 (PMC7940756; doi:10.3389/fimmu.2021.611366)

Supplementary Figures

Table S1 Correlation of IL-6 and CRP to CRS

	Pearson Correlation	
	Rho	*P*
CRS *vs* CRP	0.379	0.000
CRS *vs* IL-6	0.498	0.000

Table S2 Serum maximum value of IL-6 and CRP related to cytokine release syndrome (all patients, n=142)

**Variable**	**CRS Grade**			**Univariate Analysis**[a]	**Multivariable Analysis**		
	**non-CRS**	**Grade1-2**	**Grade3-5**	***P* value**	**OR**	**CI (95%)**	***P* value**
IL-6 during CRS,							
Max, Median [IQR]	14(6.07-43.79)	78.86(26.16-379.82)	380.45(58.01-1668.75)	0.000	1.001	1.0-1.001	0.000
CRP							
Max, Median [IQR]	41.3(7.34-78.40)	81.4(31.5-153)	131.35(57.73-200)	0.000	1.011	1.005-1.017	0.000

*****Two-sided P-values calculated based on Kruskal-Wallis test for continuous variables

Table S3 Serum maximum value of IL-6, CRP and Minimum level of CD4/CD8 related to cytokine release syndrome (patients with ALL, n=55)

Variable	CRS Grade			Univariate Analysis *P* value	Multivariable Analys		
	non-CRS	Grade 1-2	Grade 3-5		OR	95%CI	*P* value
**IL-6 during CRS** Median [IQR]	16(2.7-99.5)	105(2.74-1000)	483(44-5000)	0.001	1.002	1.0-1.004	0.030
**CRP during CRS** Median [IQR]	42.3(0.8-131.6)	98.1 (2.3-257.1)	143(9-204)	0.008	1.010	1.0-1.019-	0.046
**CD4/CD8** Median [IQR]	0.79(0.09-2-29)	0.46(0.01-4.18)	0.25(0.09-1.03)	0.028	-	-	

Table S4 Serum maximum value of IL-6 and CRP related to cytokine release syndrome (patients with Lymphoma, n=62)

Variable	CRS Grade			Univariate Analysis *P* value	Multivariable Analys		
	non-CRS	Grade 1-2	Grade 3-5		OR	95%CI	*P* value
**IL-6 during CRS**,							
Median [IQR]	8.67(4.41-176.6)	55(20.8-144)	130(18.9-1884)	0.016	1.001	1.0-1.003	0.011
**CRP during CRS**							
Median [IQR]	31.2(8.5-50)	78(34.5-143)	91.8(30-198)	0.054	1.012	1.002-1.021	0.018

Table S5. Serum maximum value of IL-6, CRP and Minimum level of CD4/CD8 related to cytokine release syndrome (patients with MM, n=25)

**Variable**	**CRS Grade**			**Univariate Analysis *P* value**
	**non-CRS**	**Grade 1**	**Grade 2-3**	
**IL-6 during CRS** S				0.321
Median [IQR]	29.845(8-5099)	44.35(11.5-5148.4)	140(19.85-1400)	
**CRP during CRS**				0.334
Median [IQR]	67.9(13.3-313)	81.4 (5.4-180)	100.8(23-200)	
**CD4/CD8**				0.462
Median [IQR]	0.24(0.12-0.29)	0.39(0.06-5.46)	0.345(0.11-9.0)	

Figure S1 The overall response rates (ORR) of ALL, lymphoma, and myeloma

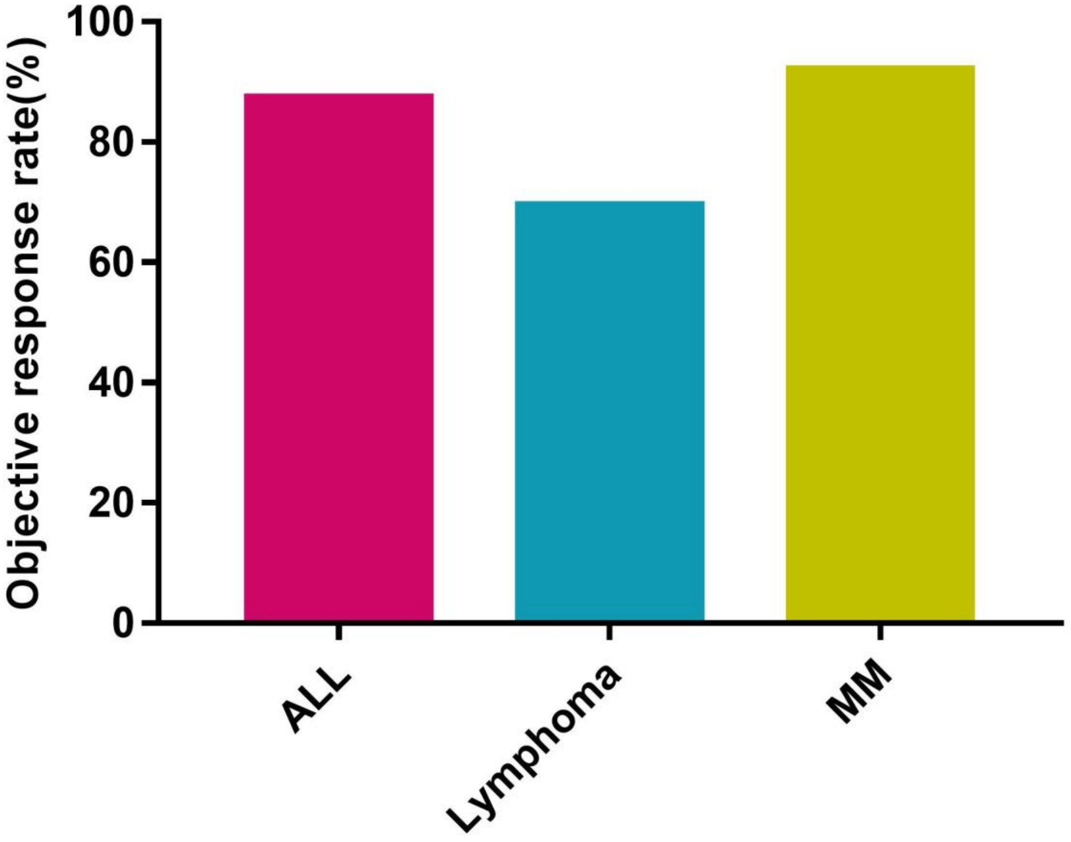

Figure S2 Changes of ferritin, IL-6, and CRP in serum

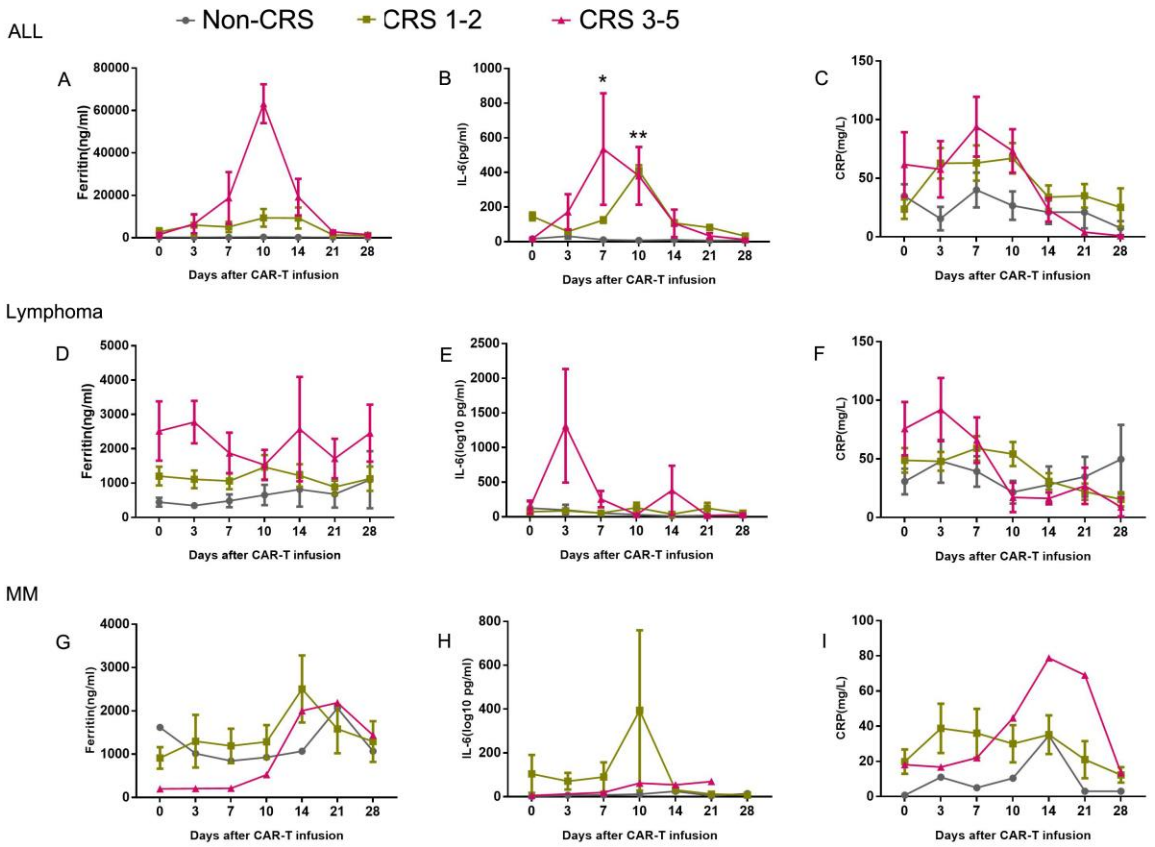

Figure S3 The sensitivity and specificity of IL-6 for occurrence of CRS

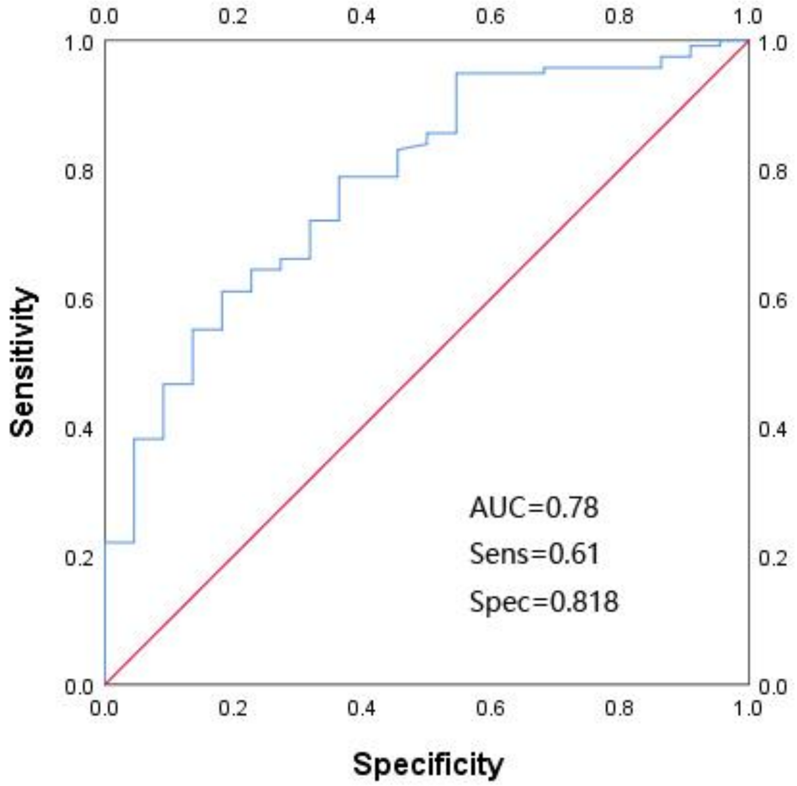

Figure S4 The sensitivity and specificity of CRP for occurrence of CRS

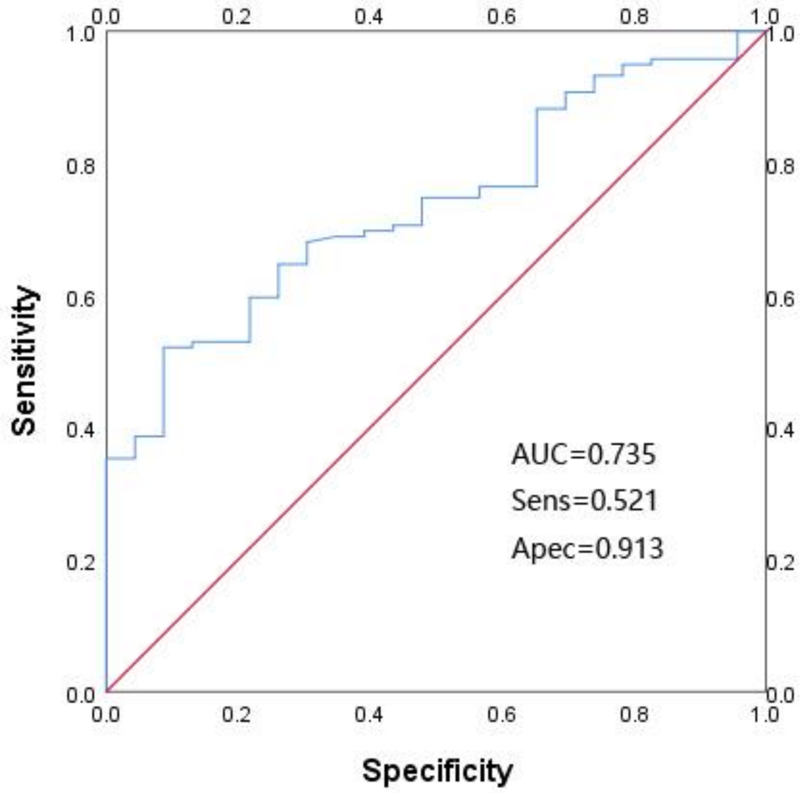

Figure S5 The sensitivity and specificity of the number of blast cell in bone marrow for occurrence of CRS

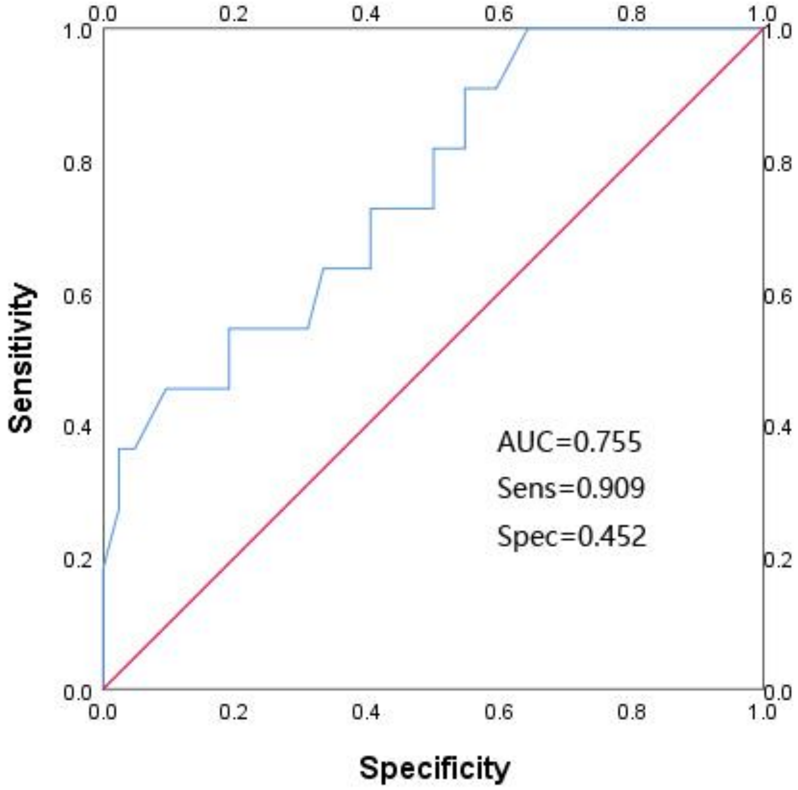

Supplement: Supplementary file 1 [file Presentation_1.pdf]
